# Sotorasib Is Not Effective in a KRAS‐Mutated Patient With Brain Metastases From Lung Adenocarcinoma due to Multiple Gene Co‐Mutations

**DOI:** 10.1002/rcr2.70206

**Published:** 2025-05-06

**Authors:** Takayuki Suetsugu, Kentaro Tsuruzono, Masashi Hatanaka, Akihiro Yamaguchi, Hajime Yonezawa, Tadashi Umehara, Keiko Mizuno, Kentaro Tanaka, Naohiko Seki, Hiromasa Inoue

**Affiliations:** ^1^ Department of Pulmonary Medicine, Graduate School of Medical and Dental Sciences Kagoshima University Kagoshima Japan; ^2^ Department of Neurosurgery, Graduate School of Medical and Dental Sciences Kagoshima University Kagoshima Japan; ^3^ Department of General Thoracic Surgery, Graduate School of Medical and Dental Sciences Kagoshima University Kagoshima Japan; ^4^ Department of Functional Genomics, Graduate School of Medicine Chiba University Chiba Japan

**Keywords:** KRAS G12C, lung adenocarcinoma, Oncomine dx target test, Sotorasib

## Abstract

A 60‐year‐old man diagnosed with lung adenocarcinoma underwent standard left lower lobectomy. However, he developed brain metastasis 10 months later, and the metastatic lesion was resected surgically. At that time, the KRAS G12C mutation was detected in the metastatic tissue by the Oncomine Dx Target Test. After 16 months, liver metastases appeared, and treatment with sotorasib was initiated. Unexpectedly, however, Sotorasib was ineffective, and the disease progressed. Cancer Genome Profiling (CGP) Test revealed that mutations of *p53* and *BRCA2* were already present in the patient's primary tumour, resulting in the initial resistance to standard targeted therapy.

## Graphical abstract

We report a case of brain metastasis that occurred during the standard post‐operative treatment for early‐stage NSCLC. Initial multiplex test was performed using the brain metastatic tissue, and the KRAS G12C mutation was detected. However, the response to Sotorasib treatment was not observed. Further genetic testing revealed mutations in multiple driver genes (*P53*, *BRCA2*, and *KRAS*) in the lung cancer cells of our patient at the time of Sotorasib initiation. We present this case as an example of the importance of genomic profiling tests in selecting the appropriate treatment in perioperative patients with driver gene‐positive NSCLC.

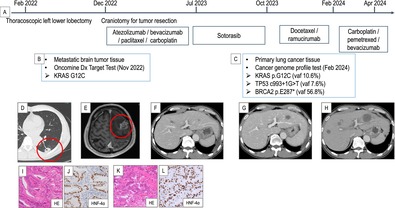

## 1 Introduction

Drugs targeting mutations in driver genes are continually being developed and are greatly improving the treatment outcomes for metastatic non‐small cell lung cancer (NSCLC) by using commercially available diagnostic tests [1]. However, such diagnostic tests cannot detect concomitant mutations potentially associated with resistance to standard targeted drugs or immunotherapies [1, 2].

We report here a case of brain metastasis that occurred during the standard post‐operative treatment for early‐stage NSCLC. Initial multiplex test was performed using the brain metastatic tissue, and the KRAS G12C mutation was detected. However, response to Sotorasib treatment was not observed. We thus performed CGP, revealing that *p53* and *BRCA2* mutations were already present in the patient's primary tumour. We present this case as an example of the importance of genomic profiling tests in selecting the appropriate treatment in perioperative patients with driver gene‐positive NSCLC.

## 2 Case Report

**FIGURE 1:**
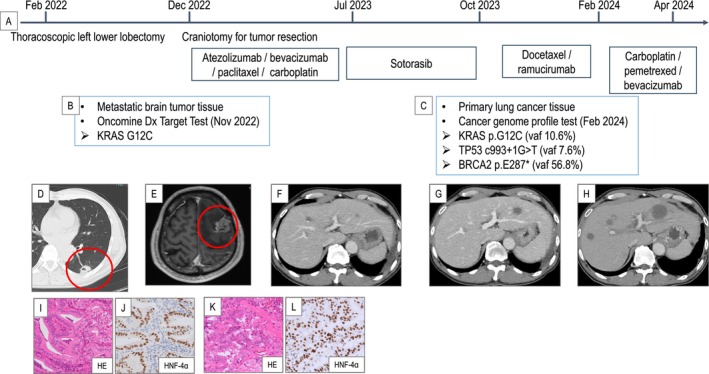
Clinical course of the patient's lung cancer treatment. (A) Timeline of the treatment course. (B, C) Results of the Oncomine Dx Target Test and a genetic profile test. (D) Computed tomography (CT) showing a 2 cm nodular shadow in the left lower lobe (red circle). (E) Contrast‐enhanced brain magnetic resonance imaging showing a lesion in the left temporal lobe (red circle). (F–H) Contrast‐enhanced CT showing progression of the liver metastasis after each treatment. (I, J) Histopathological findings of the primary tumour. Haematoxylin and eosin (HE) staining showed papillary tumour growth, and the tumour tested positive for HNF4‐α. (K, L) Histopathological findings of the metastatic brain tumour. Similar to the primary lung lesion, papillary tumour growth with positivity for HNF4‐α was observed. vaf: Variant allele frequency.

A 60‐year‐old man with a history of smoking and a Brinkman index of 360 underwent chemo‐radiotherapy for oesophageal cancer (squamous cell carcinoma, c‐T4N1M0 stage IVa) at age 43, and the patient had been under observation without recurrence. Seven years after such treatment, chest CT revealed a nodular shadow in the left lower lobe. Due to the tendency for the tumour to grow, he underwent left lower lobectomy in February 2022. The clinical course of the treatment is shown in Figure 1. He was diagnosed with p‐T2aN0M0 (stage IB) primary lung mucinous adenocarcinoma (Figure 1D,I,J). He subsequently underwent postoperative chemotherapy with uracil and tegafur, but unfortunately, the disease recurred with the development of brain metastasis 10 months after resection. A craniotomy was performed, and the pathological findings revealed papillary tumour growth positive for HNF4‐α, resembling primary lung cancer tissue (Figure 1E,K,L) Programmed death ligand 1 (PD‐L1) Tumoral Proportion Score was 10%.

This led to the diagnosis of a metastatic brain tumour from lung mucinous adenocarcinoma. The Oncomine Dx Target Test was conducted on the resected brain tissue, revealing the KRAS G12C mutation. Atezolizumab plus bevacizumab, paclitaxel, and carboplatin was administered as the first‐line treatment for the recurrent lesion, but liver metastasis appeared in July 2023. Sotorasib was started at 960 mg/day as the second‐line treatment but was reduced in two stages to 240 mg/day due to adverse events, including grade 3 elevated liver enzymes and grade 2 hypertension. However, it was completely ineffective, and no response was observed with subsequent treatments (Figure 1F,G). A CGP test was performed on the primary lung lesion before treatment, revealing mutations in *p53* and *BRCA2*.

Third‐ and fourth‐line treatments were performed, but neither regimen resulted in any tumour shrinkage (Figure 1H). Upon inquiring about the patient's family history, we discovered that his father died of lung cancer and his mother of breast cancer. The patient underwent genetic counselling, but despite our suspicion of Li‐Fraumeni syndrome and recommendation for germline testing, he declined.

## 3 Discussion

We here present a case of a patient who had relapsed after surgery. At the time of recurrence diagnosis, we found he had KRAS G12C mutation by Oncomine Dx Target Test. After 1st line treatment with combined immunotherapy, the patient was administered Sotorasib, a specific inhibitor of the KRAS G12C mutation, but it did not show any efficacy, and CGP test revealed that *p53* and *BRCA2* coexisted at the time of initiating Sotorasib. This could account for the complete resistance of such treatment.

In clinical practice for lung cancer, multiplex gene testing using next‐generation sequencing to simultaneously detect multiple driver gene mutations, such as The Oncomine Dx Target Test and AmoyDx, is the current recommendation at the time of initial diagnosis for metastatic NSCLC patients [2]. Such current tests are useful for detecting driver genes and determining treatment with molecularly targeted drugs corresponding to the detected genes; however, it has an intrinsic problem in that it cannot measure co‐mutations in genes that are essentially involved in the inhibitory effects of molecularly targeted drugs [3].

In our case, CGP was performed in the primary tumour to elucidate why Sotorasib was ineffective and to determine alternative treatments. Surprisingly, *p53* and *BRCA2* mutations were detected in the patient's primary tumour. *p53*, known as the guardian of the genome, plays a central role in regulating cell cycle progression, DNA repair, apoptosis, and senescence [4]. The wild type‐p53 protein acts as a tumour suppressor, whereas its mutated form exhibits oncogenic properties. The mutated p53 protein reduced tumour suppressive roles and induced malignant phenotypes, including drug resistance. Unfortunately, therapeutic options for TP53‐mutated cancers remain limited [4, 5].

DNA repair pathways promoted by BRCA1 and BRCA2 are primarily manifested through symmetric rejoining of DNA replication forks and double‐strand breaks. Therefore, BRCA defective‐cancer cells have been shown to be sensitive to cross‐linking agents, such as mitomycin and cisplatin [4, 5]. The subgroup of patients harbouring these mutations may benefit from cross‐linking agents.

Poly (ADP‐ribose) polymerase (PARP) enzymes are involved in the recognition and repair of DNA breaks [6]. The inhibition of PARP activity has been shown to exert antitumor effects in tumours harbouring BRCA1 or BRCA2 mutations and exhibiting homologous recombination deficiency. PARP inhibitors (e.g., olaparib, niraparib and talazoparib) are being used to treat cancers (e.g., ovarian, breast, and pancreatic cancers) with these gene mutations [6]. PARP inhibitors are a potentially promising treatment for NSCLC; however, there are no approved drugs for metastatic NSCLC, and future development is an urgent issue [6].

A key question in the current strategy of lung cancer treatment as precision medicine is when to perform CGP testing for optimising the treatment of the patient. It has become clear that some of the genes currently included as part of CGP are essentially involved in the efficacy of standard molecular targeted drugs and immunotherapies [2]. Further subtypes of patients with KRAS mutation‐ positive lung cancer are necessary. In invasive mucinous adenocarcinoma, transition mutations such as G12D and G12V are frequently observed [7] In the near future, drugs that target G12D or G12V mutations will likely be developed. We believe that the time has come for the development of a new diagnostic test that can measure these genes as an initial multiplex test, rather than as part of CGP.

In conclusion, genetic testing revealed mutations in multiple driver genes (*P53*, *BRCA2*, and *KRAS*) in the lung cancer cells of our patient at the time of Sotorasib initiation. Before starting standard treatment, it is clearly necessary to determine the genomic information of cancer cells to select the best treatment regimen for metastatic NSCLC patients.

## Author Contributions

Article manuscript preparation: Takayuki Suetsuguand Naohiko Seki. Clinical information review: Keiko Mizuno and Kentaro Tanaka. Treating patients with lung cancer: Kentaro Tanaka, Masashi Hatanaka, and Akihiro Yamaguchi. Treatment of brain metastases: Hajime Yonezawa. Overall management of patient clinical information: Hiromasa Inoue.

## Ethics Statement

We obtained informed consent from the patient described in this manuscript for publication of this manuscript and approval from the Ethics Committee on Epidemiological and Related Studies, Sakuragaoka Campus, Kagoshima University (approval no. 220099 eki, August 26, 2022).

## Conflicts of Interest

K.T. has a conflict of interest with Amgen K.K., Chugai Pharmaceutical, and Nippon Eli Lilly, but the other authors declare no conflicts of interest.

## Data Availability

Data sharing not applicable to this article as no datasets were generated or analysed during the current study.
